# Oscillatory dynamics of an electrically driven dissipative structure

**DOI:** 10.1371/journal.pone.0217305

**Published:** 2019-05-29

**Authors:** Benjamin De Bari, James A. Dixon, Bruce A. Kay, Dilip Kondepudi

**Affiliations:** 1 Center for the Ecological Study of Perception and Action, University of Connecticut, Storrs, Connecticut, United States of America; 2 Department of Chemistry, Wake Forest University, Winston-Salem, North Carolina, United States of America; Consejo Nacional de Investigaciones Cientificas y Tecnicas, ARGENTINA

## Abstract

Physical systems open to a flow of energy can exhibit spontaneous symmetry breaking and self-organization. These nonequilibrium self-organized systems are known as dissipative structures. We study the oscillatory mode of an electrically driven dissipative structure. Our system consists of aluminum beads in shallow oil, which, when subjected to a high voltage, self-organize into connected ‘tree’ structures. The tree structures serve as pathways for the conduction of charge to ground. This system shows a variety of spatio-temporal behaviors, such as oscillating movement of the tree structures. Utilizing a dynamical systems model of the electromagnetic phenomena, we explore a potential mechanism underlying the system’s behavior and use the model to make additional empirical predictions. The model reproduces the oscillatory behavior observed in the real system, and the behavior of the real system is consistent with predictions from the model under various constraints. From the empirical results and the mathematical model, we observe a tendency for the system to select modes of behavior with increased dissipation, or higher rates of entropy production, in accord with the proposed Maximum Entropy Production (MEP) Principle.

## Introduction

Developments in non-equilibrium thermodynamics have yielded an account of how physical structures spontaneously emerge from flows of energy and matter [1,2] in non-living systems. It has long been recognized that biological systems are a subset of this larger class of self-organized, nonequilibrium systems [3,4,5]. This account has clear implications for our understanding of morphological structures in biotic systems. However, biological systems also have a functional aspect; they behave in ways that allow them to maintain their own existence. Typically, function is assumed to be a higher-order property of living systems, dependent upon the pre-existence of a variety of supporting structures. We have presented evidence that dissipative structures can be end-directed in a way that is functional [6,7,8]. Specifically, dissipative structures are end-directed towards states that generate higher rates of entropy production. (In the literature the “rate of entropy production” is also often referred to simply as “entropy production”; we shall use both terms interchangeably). This end-directedness is functional for dissipative structures, because increasing the rate of entropy production in these systems seems to increase the stability of the structure, thus allowing it to persist over longer times and stronger perturbations. Our work shows that both the morphology and behavior of dissipative structures will change to increase the rate of entropy production, thus increasing the structure’s own probability of existence. In this way, maximizing the rate of entropy production appears to provide a fundamental form of functionality for dissipative structures.

### Electrical self-organized Foraging Implementation

**Fig 1:**
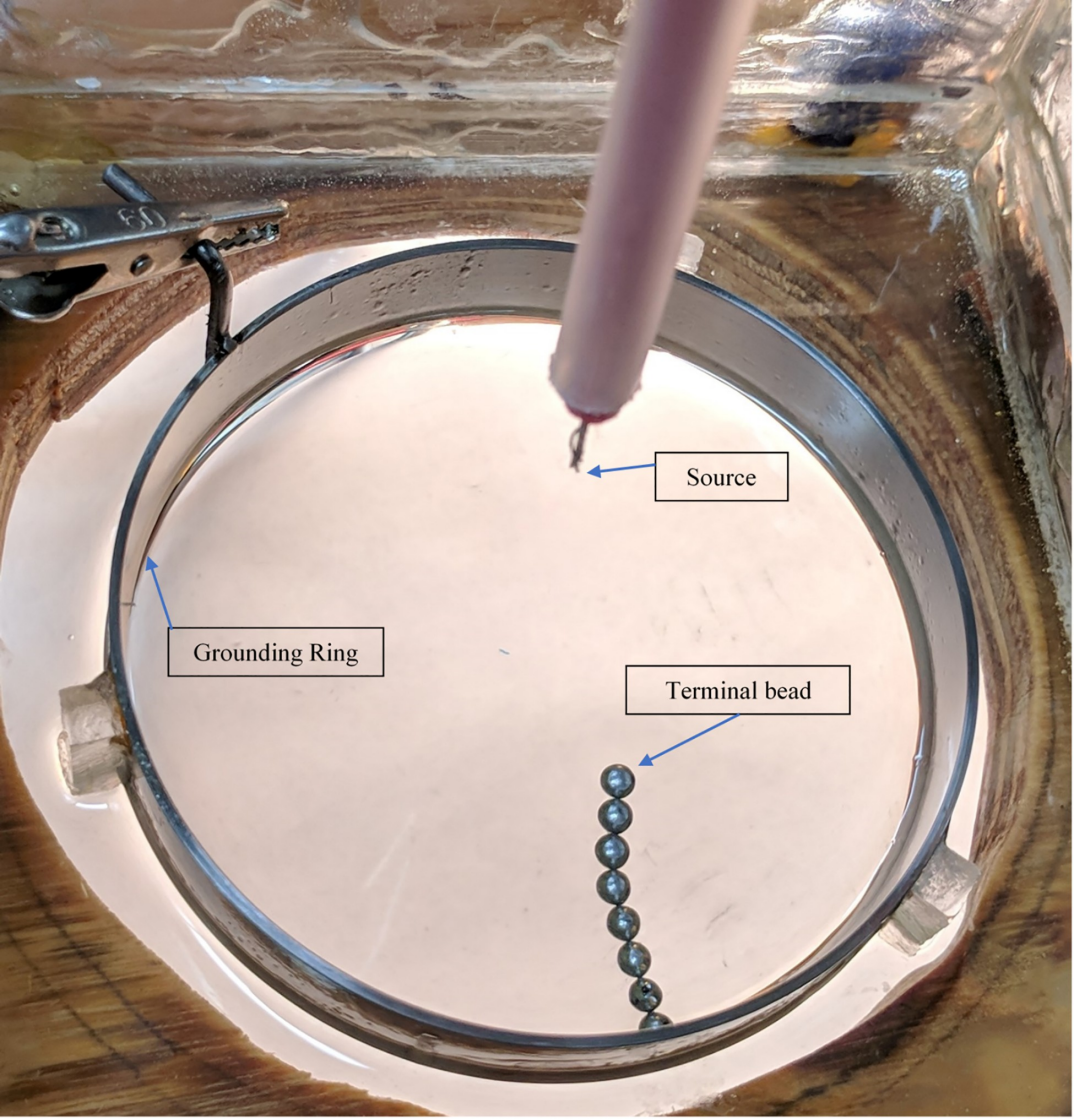
The E-SOFI. A depiction of the E-SOFI setup with a single tree of beads.

The nonequilibrium system we have been studying is driven by high voltage. It consists of conducting beads, 4.0mm in diameter, immersed in oil in a petri dish. A voltage in the range 15–30 kV is applied between a source electrode, located about 5.0 cm above the surface of the oil, and a ring-shaped ground electrode placed in the oil (Fig 1). Driven by the voltage, the beads self-organize into a tree-like structure with its trunk originating from the ring electrode. Once formed, the tree’s branches sway and the tree’s trunk itself can move along the ring electrode in complex ways. We refer to this system as an Electrical Self-Organized Foraging Implementation (E-SOFI). For detailed exploration of the system’s properties, see [8].

The formation of the trees can be understood as a consequence of the electrodynamic forces in the dish, whereby the imposed field drives the dipolar beads to connect to ground and conduct the charge away. We can understand the trees within the E-SOFI system as dissipative structures because they are supported by the dissipation of energy. Consistently we find that once the trees form, the rate of entropy production increases dramatically [7], a definitive characteristic of dissipative structures. Hubler and his colleagues [9] investigated the morphological properties of a very similar electroconductive dissipative system, finding that the system evolved towards statistically similar distributions over variable numbers of elements, all of which tended towards states of low electrical resistance; states promoting the dissipation of charge. Given the prominent place of entropy production in the theory of dissipative structures, we have focused on the rate of entropy production as the key variable. The rate of entropy production Σ can be calculated for the system as a function of the voltage V, the current I, and the temperature T (Eq 1). The voltage is held constant within trials, and the temperature is effectively constant within trials (i.e. there is only negligible heating due to the system running). The current depends on the position of the tree structure X and the velocity field of the oil V.

$$\Sigma \;=\;\frac{d_{i}S}{dt}\;=\;\frac{V*I(x,v)}{T}$$ (1)

Following their development, the trees will tend to move in the dish, exhibiting oscillations of the tip of the tree, as well as the whole tree moving along the ring in the dish. From the perspective of maximizing the rate of entropy production, oscillatory activity of the trees is a bit surprising, since it seems likely that one location in the dish would yield the maximal rate of entropy production (most likely directly below the source electrode).

**Fig 2:**
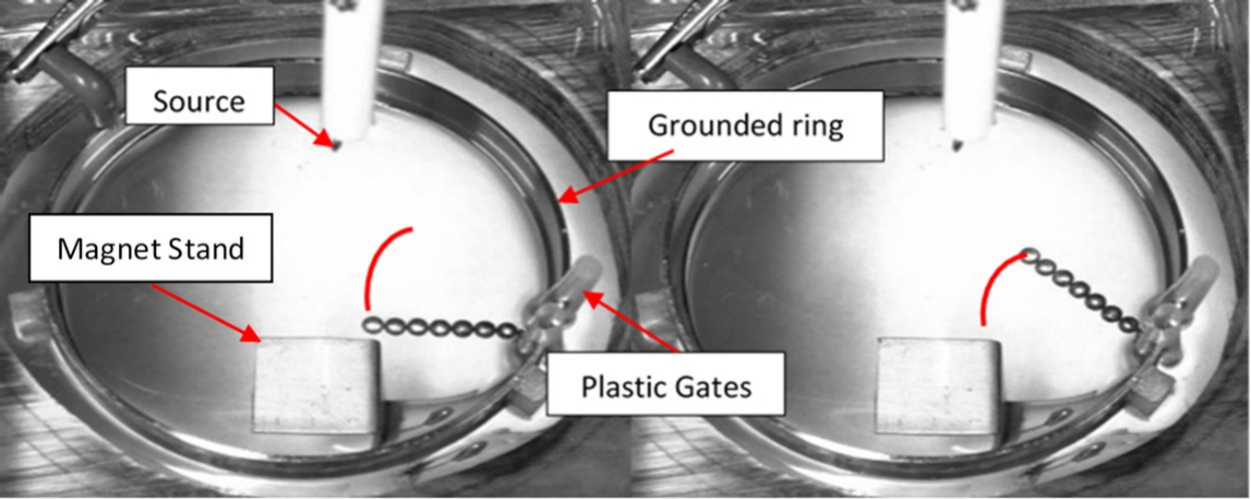
Oscillatory trajectory of E-SOFI tree. A single tree is constrained at the base by plastic gates. While the system is running, the tree will tend to swing back and forth in an approximately one-dimensional arc. The metal plate below the dish is the surface for placing a magnet in later experiments.

One possible explanation was that the oil flow around the tree was creating the oscillations, but our observations of oil flow, using non-conducting micro-beads, suggested that the oil flow was not substantially involved. Another possibility, that we pursue in detail here, was that the forces driving the oscillations may come from charges collecting on the oil surface. One can readily observe the charges impacting the oil surface (e.g., gentle waves are observed when sufficient voltage is applied). For a variety of setups, the tree will tend to oscillate on its base bead, swinging back and forth (Fig 2). Similar oscillatory behavior in a self-organized electrodynamic system was observed by Belkin et al., which evolved towards a state of increased entropy production, and exhibited quasi-periodic mechanical activity [10]. In chemical systems, oscillations similarly ensue when the steady state becomes unstable [1].

In previous work, we found that when a tree is constrained to remain in a region of low concentration of charge (i.e. displaced far from the source), and then released, it will reliably traverse the dish to find regions that support higher rates of entropy production [6]. These results suggest that the tree may be end-directed to move in such a way that increases Σ. We suggest that similarly, the oscillatory mode of the E-SOFI and the instability of the steady-state is a result of the system tending towards configurations that increase Σ.

### The charge-depletion model (CDM)

The primary motivation for the model was to elucidate why the tree, given constant constraints in the geometry of the dish and applied voltage, should so readily be driven to dynamic behavior? As a potential explanation of the tree oscillations, we consider that wherever the tree is, it conducts charges away from that region, delivering it to ground. For a given region local to the tree, if the rate of charge depletion via the tree is greater than the rate of supply of charge by the source, a pocket of low charge density relative to neighboring regions will form. Once the tree has created a region of relatively low charge-density, it will tend to move to areas of the dish with higher charge density, towards which it is drawn due to Coulomb forces. While it sweeps through these regions, it will conduct away local charges, and charge will accumulate more in distal regions. The tree will thus tend not to settle into one region, but to move to wherever there is more charge available. We suggest that this charge depletion dynamic is the basis for the motion of the E-SOFI.

This understanding of the coupling between the distribution of charge and the location of the bead serves as the foundation for the development of the charge-depletion model (CDM). The CDM consists of two sets of differential equations, one governing the distribution of charge, the other determining the forces on the bead and its consequent trajectory through the space. The model, developed in MATLAB, operates over a one-dimensional space *x*(*i*) consisting of i = 1 to i = n(1000) discrete locations, and is represented as n+3 time-dependent ODEs (n charges, position of bead, velocity of bead, and current). The forces on the bead are derived by approximating the bead as a conducting sphere in a uniform electric field. The conducting bead at the tip may carry a charge q and acquire a dipole moment *μ* due to the non-uniform charge distribution (ignoring higher moments). For simplicity, we assume the average values for both charge q and dipole moment *μ* remain constant during the motion. The force on the bead along the line of oscillation (x axis) will depend on the field E and the field gradient $\frac{\partial E}{\partial x}\;$ with respect to dimension x. The electric forces on the bead is given by:

$${\ddot{x}}_{b}(t)\;=\;qE+\;\mu \frac{\partial E}{\partial x}$$ (2)

Our model considers a single bead in a one-dimensional space x, through which the bead can move, and over which charge accumulates. The field at the bead (*E)*, a function of the amount of charge *y*_*i*_ at each location *x*_*i*_ and the distance of the charge from the bead, is approximately:

$$E\;=\;\frac{1}{4\pi \varepsilon_{0}}\sum_{i}\frac{y_{i}}{s_{i}^{2}}{\hat{s}}_{i}$$ (3)

where *s*_*i*_ is the displacement vector between the bead and the charge. ${\hat{s}}_{i}$ behaves like the unit vector in electrostatics; here in the one-dimensional space of the model it takes the values ±1 assigning directionality to the field’s effect. In the model, the electric constant *ε*_0_ is left out, since the units are arbitrary. The force on the charged bead due to the electric field for a discrete charge distribution (Eq (4)) is modeled with parameter q representing the charge carried by the bead, and E, the electric field. The Coulomb force is the product of the charge on the bead and the electric field at the bead:

$$qE\;=\;q\frac{1}{4\pi }\sum_{i}\frac{y_{i}}{s_{i}^{2}}{\hat{s}}_{i}$$ (4)

The gradient of the field is similarly a function of the amount and location of charge:

$$\frac{\partial }{\partial x}E\;=\;\frac{1}{4\pi }\sum_{i}\frac{\partial }{\partial x}\frac{y_{i}}{{s_{i}}^{2}}{\hat{s}}_{i}$$ (5)

In the one-dimensional space of the model the gradient of the field can be approximated as the difference between values of the field at adjacent points. The gradient of the field at the bead is calculated as the average of the gradient immediately adjacent to the bead. The gradient of the field to the right of the bead,∇*E*_*r*_, is the difference between the field at the adjacent cell (*x*_*b*_ + 1), and the field at the bead’s position (*x*_*b*_), and similarly for the gradient to the left (Eqs 6 & 7). The gradient at the bead is taken as the average of the gradient to the right and that to the left.

$$\nabla E_{r}\;=\;E(x_{b}+1)-\;E(x_{b})$$ (6)

$$\nabla E_{l}\;=\;E(x_{b}-1)-\;E(x_{b})$$ (7)

$$\nabla E_{b}\;=\;\frac{\;\nabla E_{r}+\;\nabla E_{l}}{2}$$ (8)

The field gradient at the bead ∇*E*_*b*_ then is positive when the field is stronger to the right, and negative when the field is stronger to the left, forcing the bead in the direction of the stronger field. The dipole moment on the bead is assumed to be constant and is approximated by parameter *μ* in the model. Additionally, we add a damping parameter *β* to represent the viscous force of the oil, multiplied with the velocity of the bead. All together the forces on the bead are modeled as:

$${\ddot{x}}_{b}(t)\;=\;-\beta {\dot{x}}_{b}+\;\mu \nabla E+qE$$ (9)

**Fig 3:**
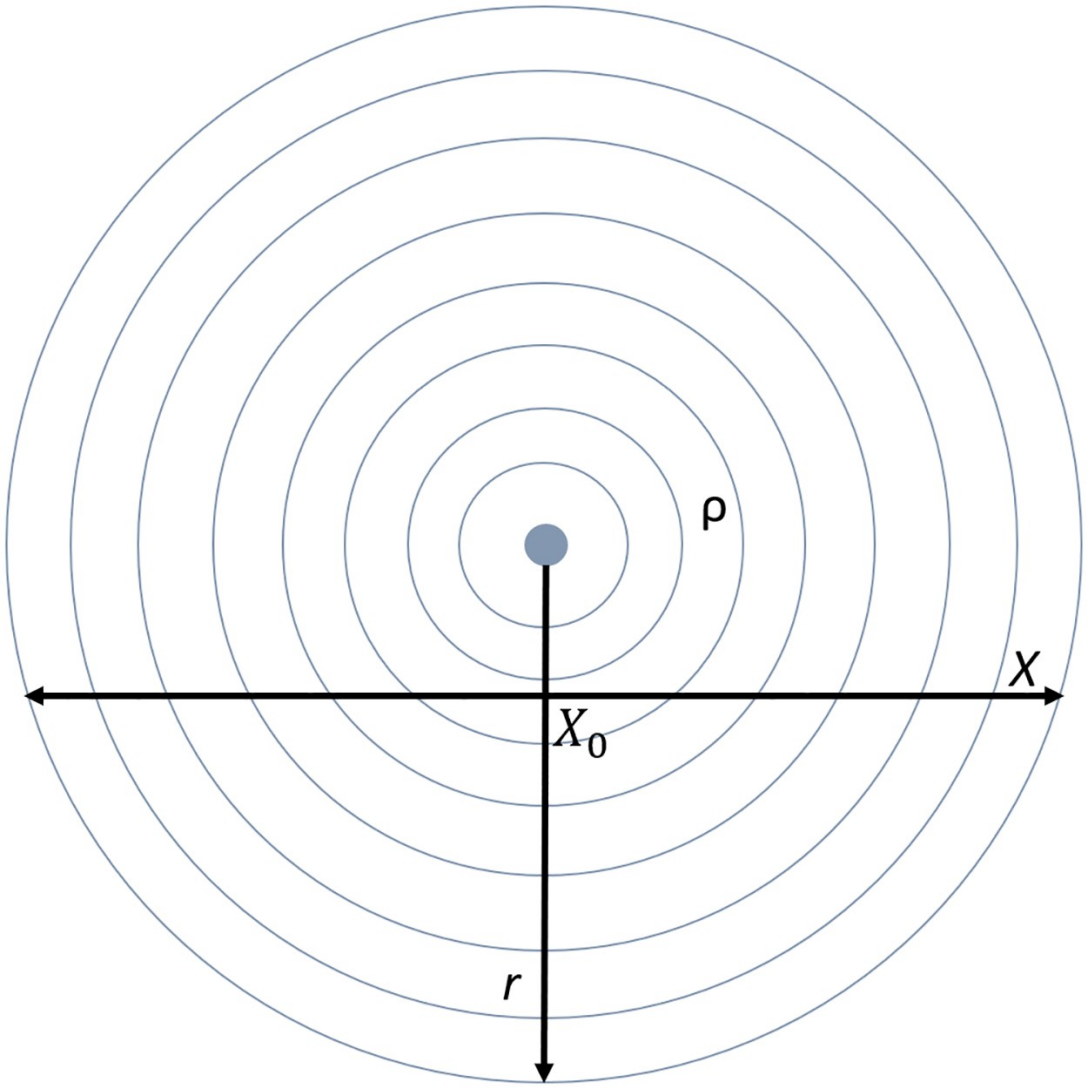
Approximating the steady-state charge distribution. The charge density varies as the inverse square of the distance from the source, r. The model takes place over a one-dimensional sample of this charge-distribution, x.

The system is approximated as a discrete one-dimensional space x, so we treat the charge distribution similarly by attributing a certain amount of charge *y*_*i*_ to each location *x*_*i*_ in that space. The amount of charge at each *x*_*i*_ is assumed to have a maximum limit *Cmax*_*i*_ which is a function of its location relative to the source and the applied voltage, describing a steady-state where the flow of charge from the source is balanced by the dissipation of charge to ground. The density of charges varies with the inverse square of the distance from the source, r (Fig 3). For the model, we approximate the charge-distribution along a one-dimensional sample x through the electric field, which will be some function of r. For modeling purposes, the *Cmax*_*i*_ along this line x is approximated as a simple function of the inverse square of the distance from the midpoint of the line (the minimum distance from the source), *x*_0_, plus a constant *Cbase* This results in a maximum of the distribution in the center of the x-space. *Cbase* sets the baseline amount of charge, akin to a voltage parameter.

$${Cmax}_{i}\;=\;\frac{1}{{\left(x_{i}-x_{0}\right)}^{2}}+Cbase$$ (10)

The supply of charge *I*_1_(*i*) to each location is a function of the applied voltage, and the difference between the amount of charge *y*_*i*_ and the maximum *Cmax*_*i*_; i.e. when there is relatively little charge, charges will accumulate more rapidly, and when the location is nearly saturated the charges accumulate at a lower rate. This assumption rests on the result in electrostatics that the work required to move charges together depends on the amount of charge; greater charge density requires greater forces for more charges to accumulate, and charge thus accumulates less readily.

The flow of charge to each point is additionally a function of the applied voltage V and the resistance R of the air and oil, both of which are taken to be constant within trials, since the voltage is computationally controlled, and temperature is effectively constant. They are thus represented as the parameter σ ~ V/R

$$I_{1i}\;=\;\sigma ({Cmax}_{i}-\;y_{i})$$ (11)

The bead conducts some fraction c_1_ = (0,1] of the charge *y*_*i*_ at each *x*_*i*_. The amount of charge depleted from each *x*_*i*_ is a function of the relative location of the bead; charges closer to the bead will be depleted more readily than charges farther away. We assume that the depletion function *I*_1_(*x*) is inversely proportional to r, the square of the distance between *x*_*i*_ and *x*_*b*_.

$$I_{2}(x)\;=\;\frac{c_{1}*q_{i}}{r^{2}}$$ (12)

$$I_{2}(x)\;=\;-\;\frac{c_{1}*y_{i}}{{\left(x_{b}-x_{i}\right)}^{2}+\;c_{2}}$$ (13)

**Fig 4:**
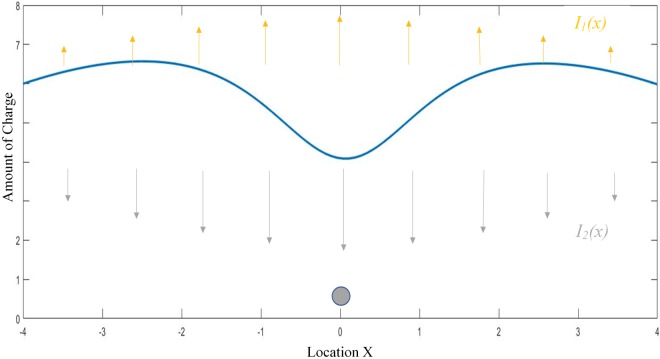
Charge distribution. The charge-distribution (blue curve). Charges accumulate according to *I*_1_(x) (orange arrows) and are depleted according to *I*_2_(x), a function of the position of the bead.

Here *c*_2_ is a constant which prevents the denominator from going to zero, and sets the maximum rate of depletion. The influence of supply and depletion of charge on the distribution is represented in (Fig 4). Putting it all together we have the rate of change of charge modeled as:

$$\dot{y}(i)\;=\;-\frac{c_{1}*y_{i}}{{\left(x_{i}-x_{b}\right)}^{2}+c_{2}}+\;\sigma ({Cmax}_{i}-y_{i})$$ (14)

The model runs via numerical simulation of the differential equations for the charge distribution (Eq 13) and the forces on the bead (Eq 8) with a Runge-Kutta style integrator. For each timestep, the charge-distribution y(x) is updated as a function of the location of the bead according to (Eq 13). The force on the bead is then calculated according to (Eq 8), and the subsequent velocity and position are updated. This process is iterated over a prescribed length of time, outputting the values for the charge distribution y(x) and the position and velocity of the bead at each time step.

This manuscript details four sets of experiments investigating the oscillatory mode of the E-SOFI. The first consists of letting a single tree oscillate while constrained at the base and analyzing the relation between the terminal bead’s activity and the current. Experiments 2, 3, and 4 were based on results from the CDM, from which we made predictions about, and tested, the behavior of the E-SOFI for each manipulation. Experiment 2 examined the oscillation frequency of the tree for varying voltage levels, for both the E-SOFI and the CDM. The third experiment compared the current through the system for two conditions, the tree freely oscillating and the tree fixed near the source via a magnet constraint. The final experiment investigated the dynamics associated with the introduction and removal of a magnetic constraint on the tree. All four experiments were analogously conducted with the CDM.

## Materials and methods

In order to investigate the nature of the tree’s motions, a minimal case of the tree oscillation was observed wherein we restricted the location of the base bead of the tree (i.e. the bead in contact with the ring electrode), such that it could only move minimally on the ring (Fig 2). This fixes the point of rotation of the tree and allows us to reduce the oscillations to one dimension. A full account of the tree motion would be complex and involve the fluid dynamics of the oil, but by restricting the motion of the tree, some of the fluid-driven motion may be reduced and the charge-driven motion may be studied. The setup is similar to that in Fig 1, with the addition of plastic insulators used to gate the tree (Fig 2). To capture the behavior of the tree, we focus primarily on the terminal bead, which swings back and forth in approximately a one-dimensional trajectory. Under constant voltage, the tree is observed to settle into steady oscillations, over trials as long as eight hours. Given the importance of the current to the systems state-selection, the purpose of this first experiment was to quantify the relationship between bead position and current.

The E-SOFI is prepared with a single tree, constrained near the base as in Fig 2. The E-SOFI is run by a Simulink model in Matlab that controls a power source supplying charge to the circuit, which maintains a voltage of approximately 20 kV between the source electrode and grounding ring. The current through the grounding electrode is collected by an analog-to-digital converter and recorded in Matlab. The video camera and the Simulink model are triggered simultaneously so that the video and current data are synchronized. The position data from the E-SOFI is collected from videos of the trials. The tracking is done via deep-learning software DeepLabCut [11], which produces x-y coordinates of the terminal bead in the tree.

Analogous to experiment 1 with the E-SOFI, the CDM is run for 2000 timesteps, calculating the position of the bead, the charge distribution at each timestep, and the current through the system. The CDM parameters for this test are in Table 1. The relative phase of the bead and current cycles are calculated via peak-picking as with the E-SOFI data.

**Table 1:** Parameters for bead oscillation in CDM.

Parameter	Value
β	2
μ	1
q	-1
C_1_	1
C_2_	1
σ	1
Cbase	15

Experiment 2 investigated the system dynamics for varying voltage levels. The E-SOFI was run for seven five-minute trials, at voltage levels varying from 16–26 kV. Oscillation rates for the bead in the E-SOFI were collected from video data.

We used the CDM to investigate this voltage-frequency relationship as in experiment 2 with the E-SOFI. The CDM is run for trials of arbitrary length, for five levels of *Cbase*. The CDM parameters for this test are in Table 2. Oscillation rates for the CDM were calculated as oscillations per time-step.

**Table 2:** CDM Parameters for oscillation rate tests.

Parameter	Value
β	1.5
μ	1
q	-1
C_1_	1
C_2_	1
σ	1
Cbase	10–30

Previous work has suggested that the E-SOFI is end-directed towards maximizing the rate of entropy production [6,7,8]. We have suggested that the oscillations observed are similarly in the service of increasing the rate of entropy production. Experiment 3 investigated this question; we compared the average current through the system in two conditions; oscillating freely, and magnetically locked at a position minimally displaced from the source electrode. We expect that the oscillatory mode should demonstrate, on average, higher current than the magnetically locked case. The E-SOFI is run at constant voltage of 16 kV, alternating five minute periods of freely oscillating and magnetically locked. While oscillating, the magnet is approximately 5.4 cm below the bottom of the dish (a distance at which it has no effect on the system), and while locked, the magnet is raised to approximately 0.2 cm below the bottom of the dish. While locked, the terminal bead of the tree is static, and the constituent beads are static or nearly static; some flexing of the tree is observed, but it maintains its integrity.

To simulate experiment 3, we compared the CDM’s current when the tree is freely-oscillating versus magnetically locked. This can be accomplished analogously in the CDM by introducing a new force to the bead’s acceleration equation (Eq 8). The magnetic force m is inversely proportional to the square of the distance between the magnet and the position of the bead. *m*_0_ is the base magnet strength, *x*_*m*_ is the position of the magnet. *c*_2_ functions as before to prevent the denominator from going to zero.

$$m\;=\;\frac{m_{0}}{{{(x}_{b}-\;x_{m})}^{2}+c_{2}}$$ (15)

The magnet may be placed at any point in the x-space. As in the E-SOFI trials, the magnet is positioned near the source at the midpoint of the oscillations, akin to the minimal displacement from the source.

$${\ddot{x}}_{b}\;=\;-bv_{b}+\;\mu \frac{dy}{dx}+qE+m(x_{b})$$ (16)

The CDM is run with the magnet parameter on, and off, for 3000 time-steps each. The average current over the damped and undamped periods is calculated as the mean of the absolute value of the current. The CDM parameters for this test are in Table 3.

**Table 3:** CDM Parameters for mean current tests.

Parameter	Value
β	1
μ	1
q	-1
C_1_	1
C_2_	1
σ	1
Cbase	15

The magnetic constraint offers another manipulation, utilized in experiment 4. We lock the bead magnetically as before to remove the oscillations, and then observe the behavior of the bead initially following the release of the magnetic constraint. Given the charge-distribution’s dependence on the location of the bead, we expect that limiting the activity space of the tree will lead to greater build-up of charge in the regions the tree does not traverse. This increased charge-gradient should have consequences for the dynamics of the bead. Following the removal of the magnet, we expect a “rebound effect”; an increase in the velocity of the bead relative to its usual velocity during unconstrained motion.

The E-SOFI is run at constant voltage of 19 kV. The tree in the E-SOFI is constrained at the beginning of the trial with the magnet, at a position near the typically extremal point of the oscillatory trajectory (i.e. nearly maximally displaced from the midpoint). The magnet is on a motorized stand allowing for its distance from the dish to be adjusted through Matlab. As in experiment 3, while the tree is locked, the magnet is approximately 0.2 cm from the bottom of the dish. While the tree is unlocked, the magnet is approximately 5.4 cm from the bottom of the dish. The rebound trials consist of an approximately one hundred-second-long period of the tree being locked, followed by the removal of the magnetic constraint, and then allowing the tree to oscillate freely for approximately two minutes. The rebound velocity is the peak velocity of the motion immediately following the removal of the magnetic constraint. The peak velocities of the cycles following are then averaged to obtain an estimate of the unconstrained velocity.

For an analogue of experiment four, we run the CDM with the bead magnetically locked (via the same force introduced for experiment three) for 1000 timesteps, then release the magnet and measure the peak velocity of the bead’s motion immediately following the release. The CDM parameters for this test are in Table 4. All data from all experiments and simulations are made available as supporting information to this manuscript, S1–S9 Datasets.

**Table 4:** CDM parameters for rebound tests.

Parameter	Value
β	1.5
μ	1
q	-1
C_1_	1
C_2_	1
σ	1
m	50

## Results

**Fig 5:**
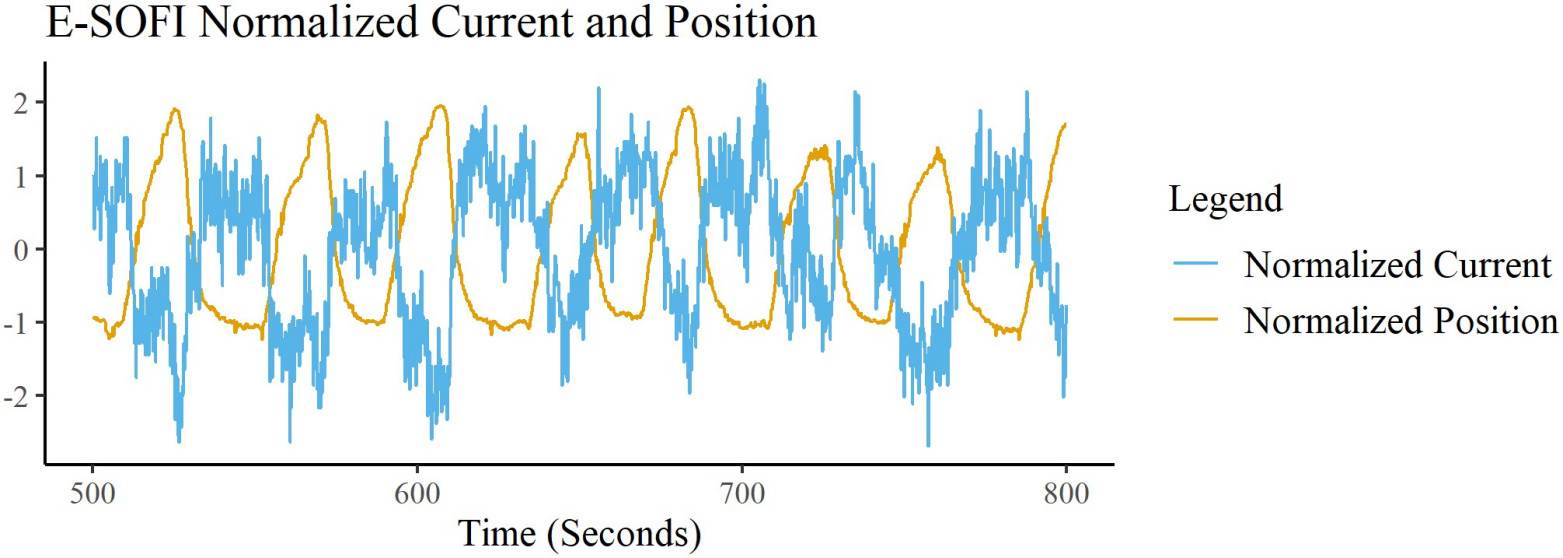
E-SOFI current bead cycles. Current and Bead displacement of the E-SOFI from a subsection of a trial. Bead displacement is distance from the source; minimum distance from source is minimum of signal, not zero-crossing. Both time-series are normalized so they appear on the same scale and the relative phase is more readily apparent. The current reaches higher values while the bead is nearest the source.

Experiment 1 was concerned primarily with the relationship between the current and the oscillations. Fig 5 shows the data from 300s of a single trial. The blue curve is the normalized displacement of the terminal bead from the source over time, and the orange curve is the normalized current through the system. The minima of the bead displacement curve correspond to the bead being minimally displaced from the source. The maxima correspond to the ends of the trajectory. A peak-picking function finds the maxima and corresponding time indices of each time-series (filtered with a net fourth-order Butterworth low-pass filter), which are then used to calculate the relative phases of the peak events. The average relative phase (current peaks in cycles of position) of the two signals is -2.7921 radians (SD = 0.7881 radians). The two signals are nearly perfectly out of phase; i.e. the current is maximal while the bead is minimally displaced from the source.

**Fig 6:**
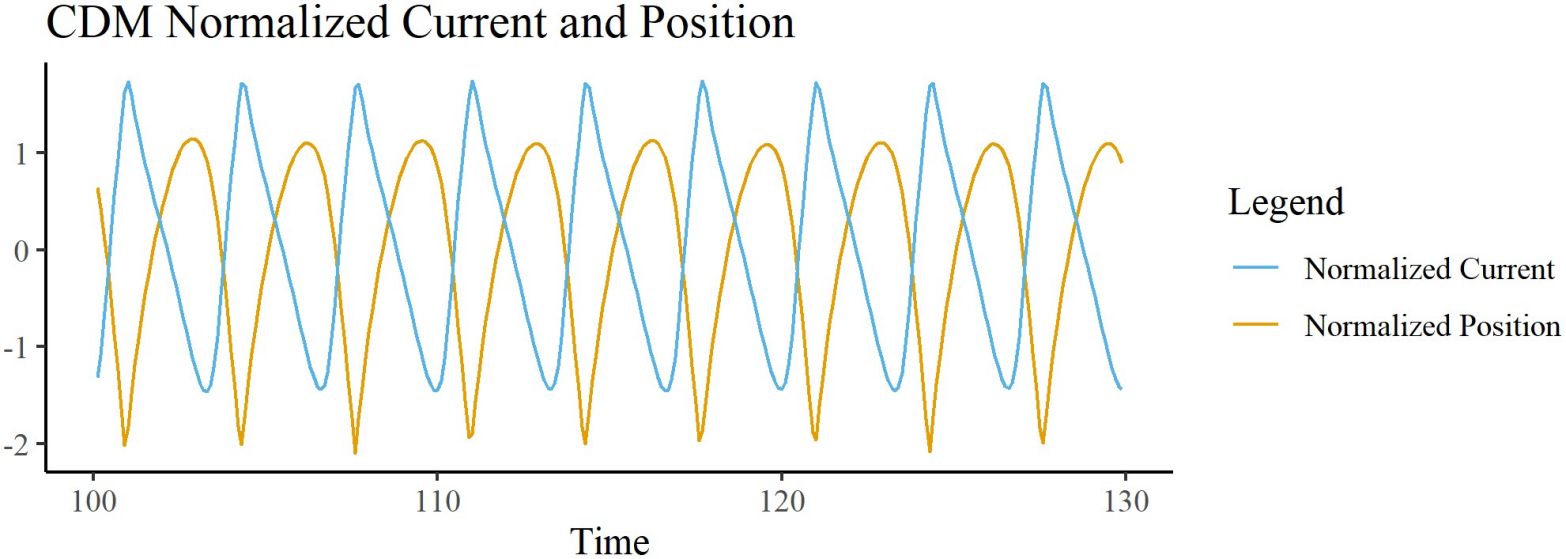
CDM bead position and current. Time-series for the position of the bead and the current over the simulation. Both time-series are normalized as in Fig 3. The orange curve is the bead’s distance from the source, with minimum distance being the minimum of the curve (not the zero crossing). The peaks of current are out of phase with the peaks of bead displacement, as in the E-SOFI.

In the CDM, current is calculated by the depletion term (negative term in Eq 14) multiplied by the integration step. Since the depletion term represents the charges leaving the charge-distribution at each point in time, we take this to be representative of the current through the ground, akin to charges leaving the system. The CDM generates oscillations of the bead relative to the source (midpoint of the x-space). The relative phase of the current and bead position is calculated via peak-picking. Current reaches a maximum almost out of phase. (M = -2.7998 radians, SD = 0.0674 radians) with the bead being minimally displaced from the source (Fig 6), similar to that observed in the E-SOFI.

**Fig 7:**
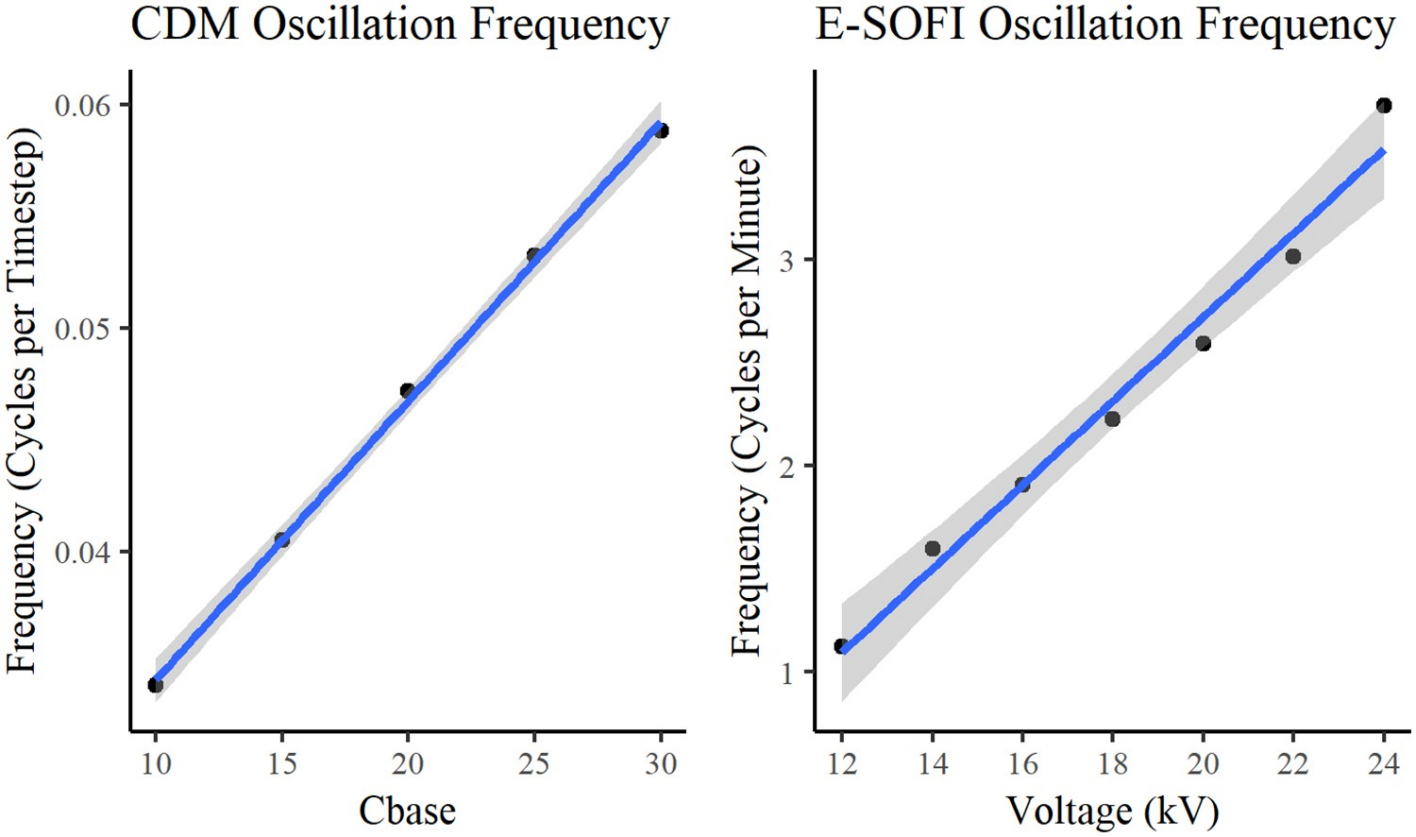
Oscillation frequency as function of voltage. [A] Bead oscillation frequency for varying values of parameter Cmin in CDM. [B] Bead oscillation frequency for varying values of voltage in E-SOFI.

Experiment two tested the tree’s oscillation frequencies for varying levels of applied voltage. Both the CDM (Fig 7A) and E-SOFI (Fig 7B) present monotonically increasing oscillation rates with increasing values of sigma and applied voltage respectively.

To evaluate the results from experiment 3, we need to consider the nature of the values of current for each system, the CDM and the E-SOFI. The current in the CDM only represents the charges conducted through the bead, while the current through the ground in the E-SOFI is generated by charges flowing through all pathways (i.e. air, oil) to all parts of the grounding ring. Previous work [8] investigated the contribution of the tree to the system’s current, by adding beads to an empty dish. The tree increases the current only very slightly relative to the baseline (e.g. 0.2 microamps above a baseline of approximately 10.5 microamps). To approximate the contribution to the current in the E-SOFI just due to the oscillations, we subtract a constant approximating the amount of current generated by pathways other than the tree from the current time-series. Additionally, the units of current in the CDM are arbitrary, and have no directly scaled connection to current in the E-SOFI. To aid comparison between the CDM and E-SOFI currents, we rescale both by the maximum current of each respective time-series.

**Fig 8:**
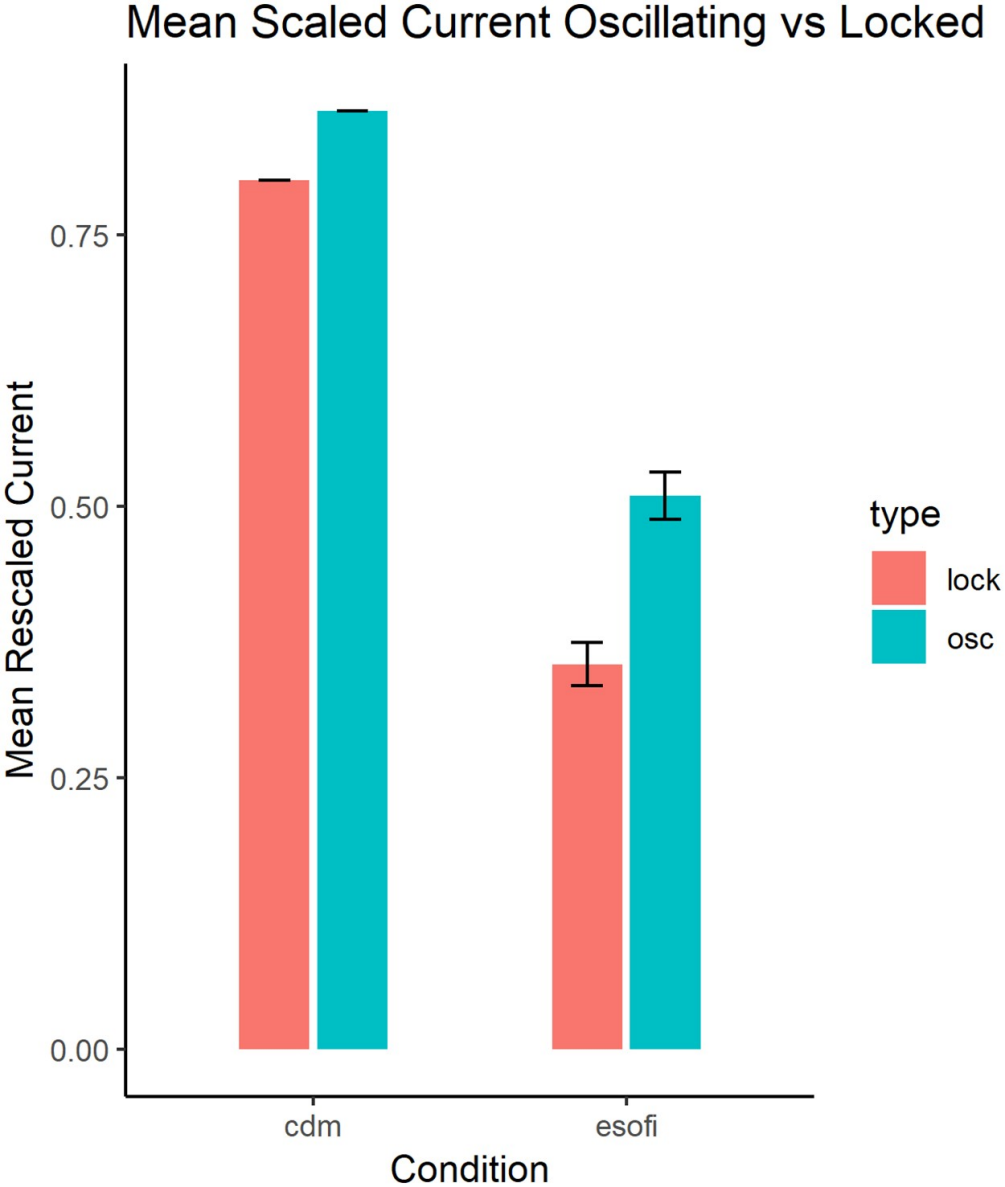
Mean currents. Mean values of the current for oscillating and locked conditions, in the E-SOFI and CDM.

Across four alternations of oscillating and locked in the E-SOFI, we find a significant difference between oscillating (M = 0.5099, SD = 0.0436) and locked (M = 0.3545, SD = 0.0395) conditions (t(3) = 5.284, p < 0.005). For the CDM, the average rescaled current while unlocked (i.e. oscillating) is 0.8642, and while locked it is 0.8001. The current is greater while oscillating than while locked via the magnet (Fig 8).

**Fig 9:**
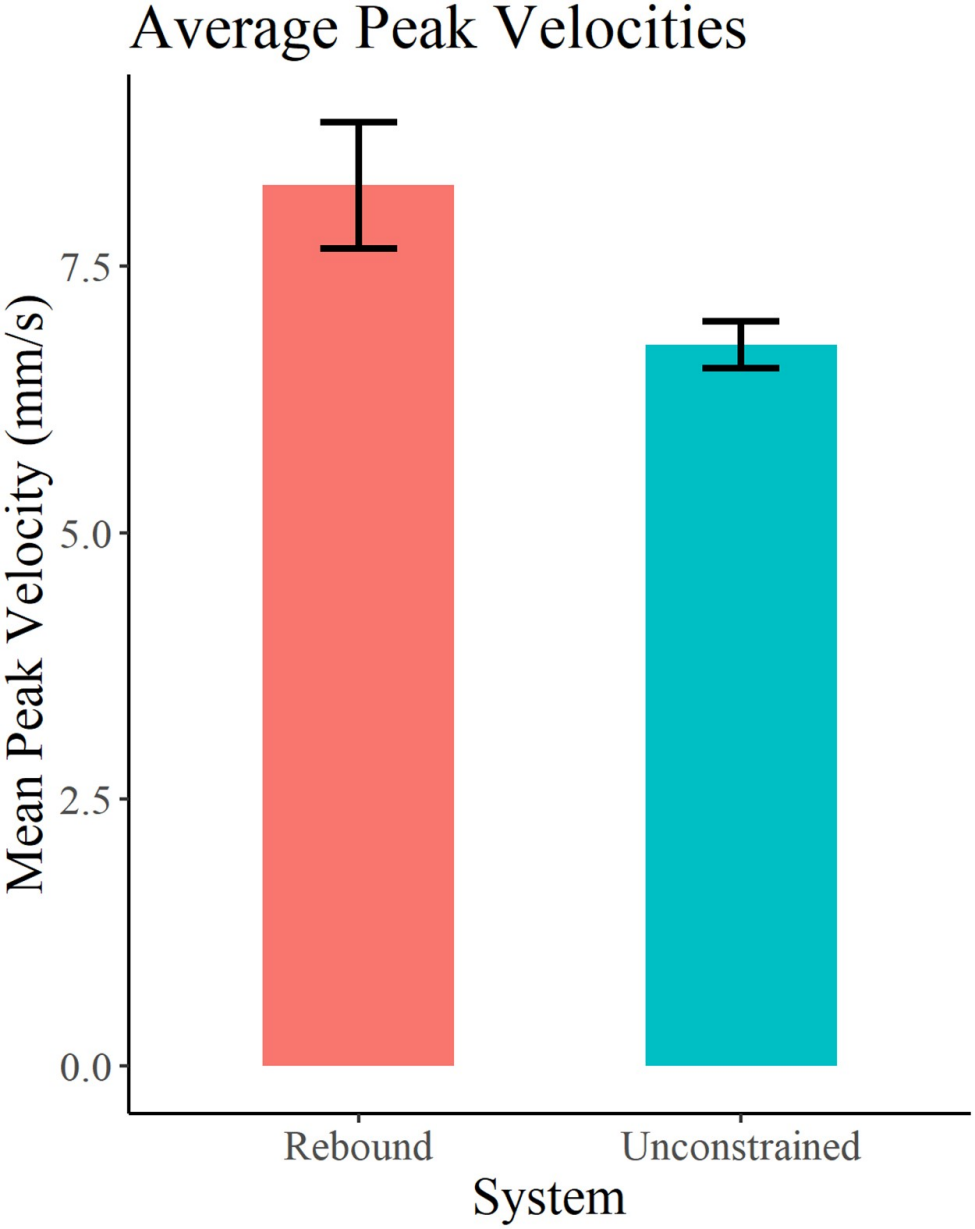
Rebound vs. unconstrained velocities. Mean rebound and unconstrained velocities from E-SOFI trials (mm/s).

**Fig 10:**
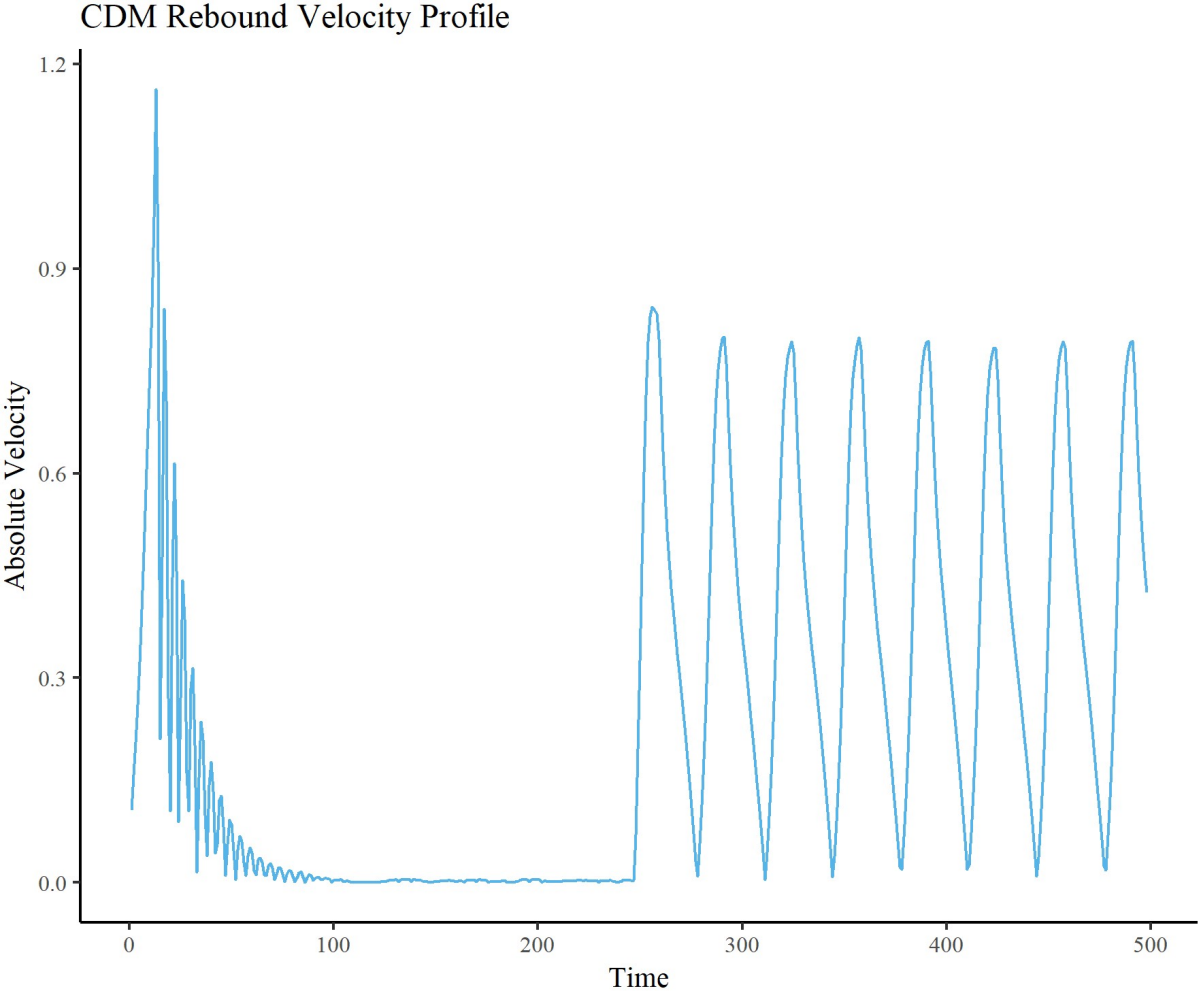
CDM rebound effect. CDM Bead velocity. Magnet is on for first 250 time-steps, and off thereafter. Velocity peaks immediately after removal of magnet, indicative of the suggested “rebound” effect. Initial velocity at beginning of time-series is due to magnetic force.

**Fig 11:**
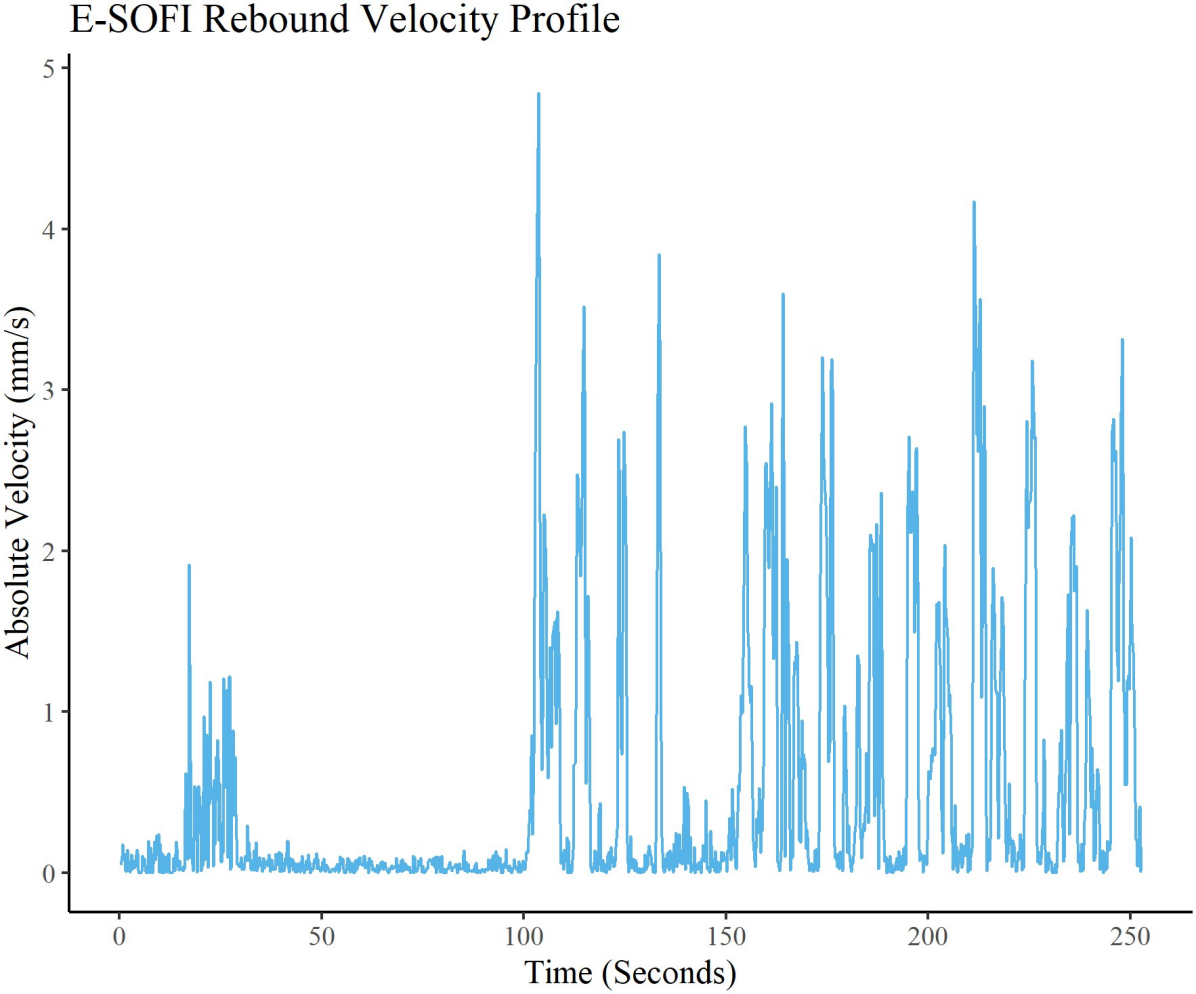
E-SOFI rebound effect. One example of the E-SOFI Rebound velocity. Release occurs at approximately 100 seconds. Peak velocity following release is the maximum of the time-series.

Velocity of the bead was computed from the tracked video coordinates. The peak velocity associated with the rebound trajectory is taken from each trial, and the remaining unconstrained peak velocities are averaged within each trial. Mean unconstrained and rebound peak velocities are shown in Table 5. A paired samples t-test showed that there was a significant (p < 0.05) difference between rebound (M = 8.256, SD = 1.568) and unconstrained (M = 6.761 SD = 0.578) conditions (t(6) = 2.3468, p = 0.047) (Fig 9). The anticipated rebound effect can be seen in the velocity data (Figs 10 and 11), wherein for both the CDM and the E-SOFI, the maximum velocity is immediately after the magnet is removed.

**Table 5:** Mean rebound and unconstrained velocities.

Rebound	Unconstrained	Trial
10.76667	6.594472	1
9.508444	6.537418	2
7.488444	7.241062	3
7.273556	5.649029	4
8.104222	7.007299	5
5.988222	6.927238	6
8.664667	7.372889	7

### Stability analysis

To explore the nature of the oscillatory mode, we test for the onset of oscillations and the instability of the steady-state as a function of the applied voltage. Dissipative structures form when the steady-state solution of a non-equilibrium process becomes unstable, and new solutions emerge [12, 13]. This bifurcation is a result of the system being pushed away from equilibrium by chemical, thermal, or electrical gradients. For example, oscillating chemical reactions occur for high reactant concentrations that prevent the system from going to equilibrium [1, 13]. The oscillatory mode in the E-SOFI similarly emerges when the system is pushed far enough out of equilibrium that the steady-state becomes unstable. Rather than an analytic solution of the CDM equations (which will be the focus of a subsequent publication), we can investigate the stability of the steady state and the Hopf bifurcation of the oscillatory mode by treating the applied voltage as a control parameter. While the model has no explicit term for voltage, changing the constant *Cbase* (in the equation for the distribution) functions analogously to changing the voltage; increasing this value increases the base amount of charge, just as an increase in voltage would.

**Fig 12:**
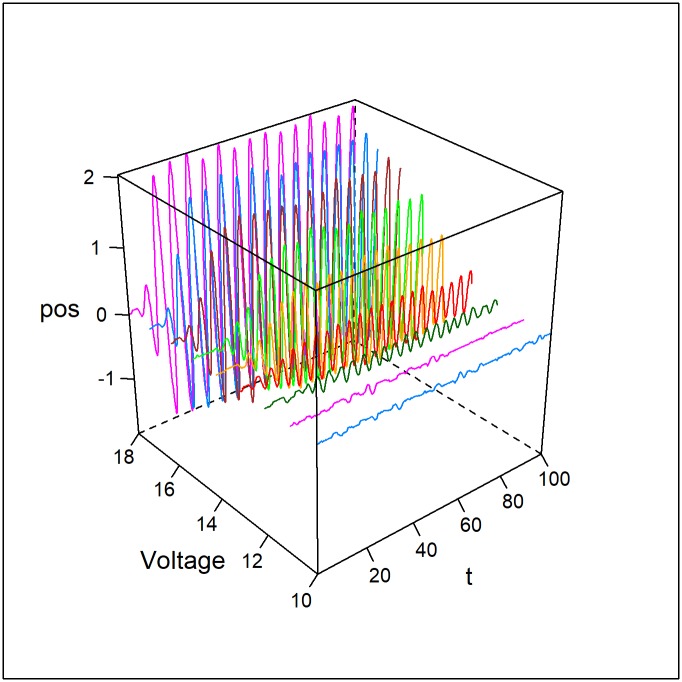
Observational stability analysis. The oscillatory mode emerges as the stable state with increasing applied voltage (Cbase). The amplitude of the oscillations increases with increasing values of the control parameter past the critical threshold.

Varying this parameter from low to relatively high values we see the onset of oscillations at a critical value, typical of a Hopf bifurcation. At the critical value, the system becomes unstable to perturbations. When the voltage is above this critical value, the system will enter into the oscillatory mode and remain there; transitions from steady-state to oscillation are readily observed. The oscillatory state is stable, as expected of a Hopf bifurcation, and the system does not transition back to a stable state. Additionally, the amplitude of these oscillations increases with the distance above the critical threshold (Fig 12).

**Fig 13:**
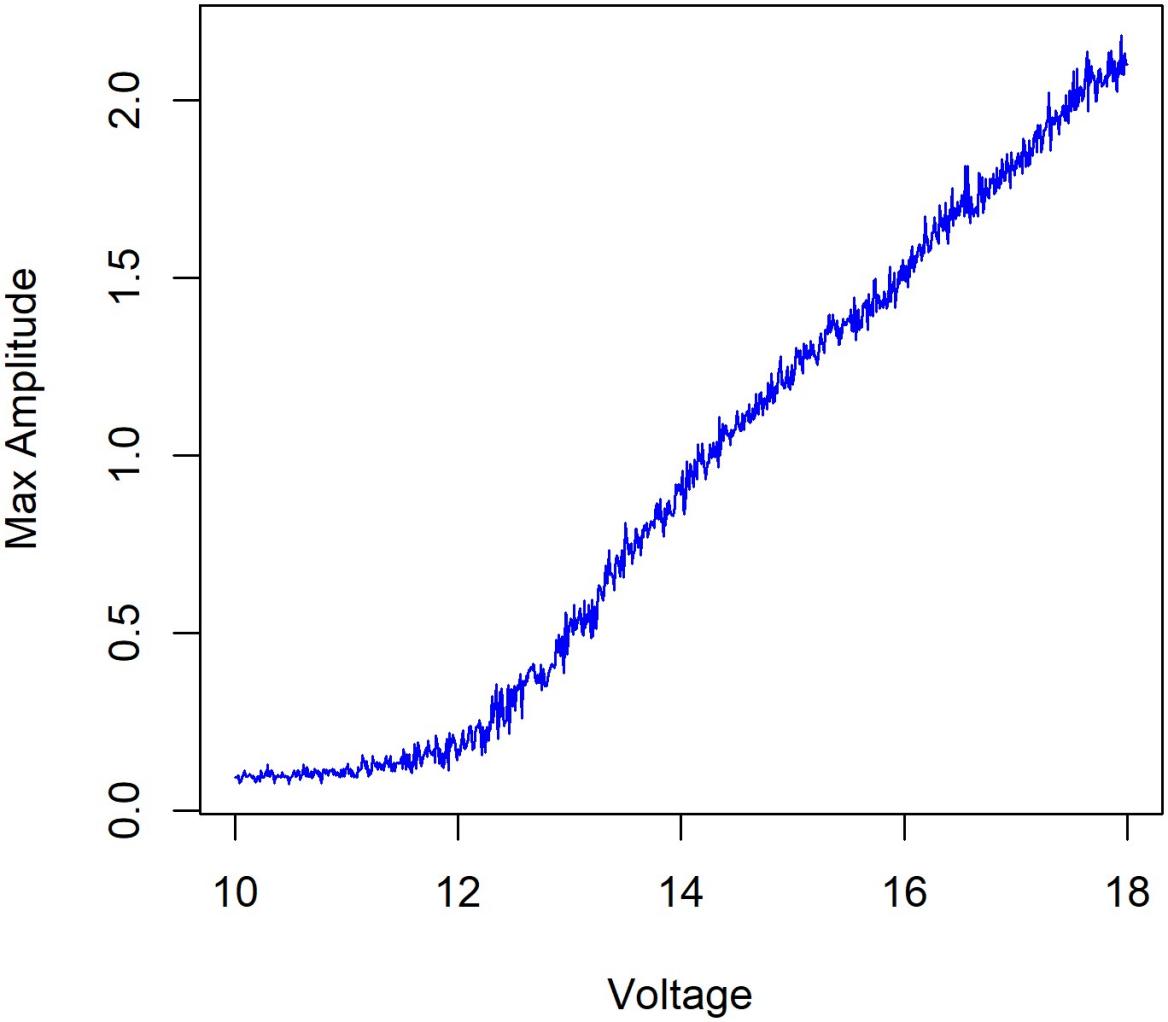
Empirical bifurcation curve. The maximum amplitude of the oscillations varies as a function of the applied voltage. Here we see that the steady-state is stable for voltage values below 12, and there are finite amplitude oscillations above this critical value, whose amplitude depends on the distance above the threshold.

Simulations were run for varying levels of applied voltage, and the mean amplitude was measured. The simulations included a noise term to ensure that the stability or instability of the steady-state was reflected. For low values of the voltage parameter, the average amplitude remains near zero, while above a critical threshold, the amplitude increases nearly parabolically, as we expect from a Hopf bifurcation (Fig 13). For the following parameters, shown in Table 6, the critical threshold of oscillations is approximately *Cbase* = 12.

**Table 6:** CDM parameters for stability analysis.

Parameter	Value
β	6
μ	1
q	-1
C_1_	1
C_2_	1
σ	1.5

## Discussion

Experiment 1 found that the cycles of the bead’s displacement and the current were nearly perfectly out of phase, meaning the current was maximal while the bead is minimally displaced from the source. These results are sensible; we ought to expect that the current is highest while the bead is nearest to the source, a region of greater charge-density. We hypothesized that the motion was driven by the build-up and depletion of charge on the surface of the oil; this mutual constraint of the charge-distribution and the bead is the core feature of the CDM. The bead in the CDM also readily oscillates. That the CDM generates oscillations similar to what is observed in the E-SOFI is a compelling result, showing that the forces on the bead as prescribed in the model capture important variables in the E-SOFI. Further the CDM data corroborate the relative phase results from the E-SOFI; we find that the current is consistently nearly out of phase with the bead reaching its maximum displacement from the source, similar to what was observed in the E-SOFI.

The oscillations appear to come about as a result of the steady state becoming unstable. By depleting charges in a local region, the bead alters the distribution of charges such that more distal regions of the dish will have higher charge density. The bead is forced in the direction of greater charge density, moves in accordance with the force, and depletes charge along the way. Charges build up elsewhere, and the bead is forced back in the other direction. The system appears to undergo a Hopf bifurcation, and the oscillations become a dynamically stable state.

Experiment 2 investigated the tree’s oscillation frequency for varying levels of voltage. For both the CDM and the E-SOFI, we observe a nearly linear relationship between the applied voltage and the frequency of oscillations (Fig 7). There is corroboration between the model and the data from the real system, supporting the hypothesized mechanism of charge-depletion.

Experiment 3 considered how the oscillations contributed to the system’s dissipation of charges, finding that the oscillatory mode for both the E-SOFI and CDM had higher time-averaged current. These results are consistent with the notion that the stability of the oscillatory mode is related to preference for states of increased entropy production. We see a tendency for the system to increase the rate of entropy production at both the short time-scale of the individual oscillations, where the tree moves in accordance with the local field conditions towards regions of higher charge-density, and the long time-scale of the system’s sustained oscillations. The selection of the oscillatory state is likely due to the amplification of fluctuations driving the system away from an unstable static steady-state, and towards a dynamically stable state of increased dissipation.

Experiment 4 observed the behavioral consequences of the introduction and removal of a magnetic constraint, revealing a rebound effect in the velocity profile of the bead. To understand this, consider that by limiting the bead’s ability to move, charges build up elsewhere in the dish to a greater degree than it otherwise would if the bead were free to access those regions. Consequently, the force imposed on the bead is greater due to this gradient, and the bead moves up that gradient more quickly than under typical unconstrained circumstances.

## Conclusions

The initial results of the relation between the current and the bead’s position, as well as the experimental confirmation of predictions generated from the CDM, suggest that the model is accurately representing some important features of the real system. The fundamental coupling between the position of the bead and the charge-distribution is well-motivated. Further development of the model should be done to attempt to capture more behaviors observed in the E-SOFI, such as results demonstrating its ability to traverse regions of low concentration of charge in order to reach regions of higher concentration of charge [7]. To improve the accuracy and scope of the model, its spatial-dependent properties should be elaborated, especially adding a second dimension. These and other developments of the CDM will enrich understanding of the behaviors of the E-SOFI system, and may have further implications for developing a principle of “maximum entropy production” or more precisely “maximum rate of entropy production” (MEP). As a formal principle, MEP has been proposed in many, sometimes seemingly contradictory forms [14, 15], but it is not a universally valid principle, applying only to a particular class of systems [14, 15, 16, 17].

To put this in perspective, we note that close to equilibrium, where thermodynamic forces and fluxes are linearly related, entropy production is minimized [12, 15]. As an example of a system in which MEP is not valid, consider a simple electrical resistor, with resistance R, across which a fixed voltage V is applied. As the current I flows, the resistor’s temperature will increase due to Ohmic heating. The rate of entropy production in this resistor d_i_S/dt = VI/R. As the temperature increases, the resistance will increase leading to a decrease in the current. Hence dS/dt will decrease until it reaches a steady state and not increase as MEP predicts. MEP rather will apply to systems for which the relations between forces and fluxes are non-linear (and likely other necessary conditions). Recent work has shown how to relate minimum entropy production and maximum entropy production, and elaborates the thermodynamic constraints and conditions for either to be the case [15]. In non-equilibrium systems, entropy is produced by irreversible processes, and if heat and other forms of energy (and matter) can flow out of the system, entropy generated in the system will flow out of the system. Precise expressions for entropy production, entropy flow and the consequent entropy balance equation for a given system can be found in texts on thermodynamics [12, 16].

We find compelling evidence that the E-SOFI is in this class of systems for which MEP is valid, and that the behavioral modes exhibited are selected in accordance with MEP [6,7,8]. We do not, presently, have an analytic description of the states of the E-SOFI, and which states are maximally dissipative. In a complex system like the E-SOFI, due to the virtually infinite number of possible configurations of the beads, it is not possible to ascertain if the rate of entropy production has reached the maximum possible value in a particular state. Hence, we can only say that our observations show that particular changes in morphology or activity lead to states of higher rate of entropy production, with the understanding that at some point the system will reach the maximum possible rate; for a fixed voltage, the entropy production has to reach a maximum value, it cannot increase indefinitely.

One conceptual roadblock to elucidating the behaviors of dissipative systems with an MEP [7,14,16] is that such a principle carries implicitly a notion of static characteristics. That is, the state in which the rate of entropy production is maximized should be obtained and then become stable. Any other state, especially time-varying states of the system, would seem to be exceptions to an MEP; if the rate of entropy production decreases (e.g. as part of an oscillatory cycle), how can the system be maximizing it? The results herein suggest the possibility that dynamic processes, and even processes wherein the rate of entropy production changes, may be driven by MEP. In the E-SOFI, the oscillatory mode, on average, results in an increased current. Moreover, the individual oscillations, wherein the tree is driven to regions of greater charge-density (and consequently greater current), suggest that the local processes supporting motion are in accordance with MEP. On both long and short time-scales the system tends to exhibit behaviors that result in increased rates of entropy production.

The dynamic (e.g. oscillatory) behavior of this, and perhaps other, dissipative systems may arise as a result of the system evolving towards states of increased entropy production, and consequently changing the nature of that end-state. In the E-SOFI this plays out through the mutual influence of the tree and the charge-distribution, creating dynamics that continually reshape the constraints under which the system seeks to increase the entropy production; dissipating local charges changes the distribution of charges, which in turn drive the motion of the tree. This coupling wherein the system manipulates its own constraints, speaks to the complexity of these dissipative systems, and perhaps the nature of function in biological systems broadly.

Organisms are similarly coupled with mutual constraint to their environment, driving the emergence of complex, coordinated, functional behaviors (e.g. collective construction of termite mounds [18]). The diversity of behavioral modes of living systems may be a result of non-equilibrium systems exploiting a variety of means for increasing the rate of entropy production [18,19]. The diverse set of dynamics which biology participates in are not, we propose, a violation of MEP; MEP need not predict only steady-states. Rather, the intimate mutuality of an organism and its environment begets dynamics which continually revise the constraints under which the system satisfies an MEP. All together, the E-SOFI presents a compelling analogue for the fundamental properties of biological behavior; end-directedness and energy-seeking, functional morphologies and behaviors supporting foraging, and a dynamic stability supported by the continual resupply of energetic resources.

## Supporting information

S1 File Charge depletion model. This file contains the equations constituting the Charge Depletion Model reviewed in this manuscript.
(M) Click here for additional data file.

S2 File Parameter selection. This file sets the parameters for the Charge Depletion Model and runs simulations.
(M) Click here for additional data file.

S3 File Integrator. This file contains a Runge-Kutta integrator used to simulate the Charge Depletion Model.
(M) Click here for additional data file.

S1 Dataset Position and current data—E-SOFI. This file is a dataset containing the raw current and position values for the E-SOFI system reported herein.
(CSV)

S2 Dataset Position and current data—CDM. This file is a dataset containing the position and current values derived from the simulations of the CDM reported herein.
(CSV)

S3 Dataset Oscillation frequency. This dataset contains the estimates of oscillation frequency for both E-SOFI and CDM.
(CSV)

S4 Dataset E-SOFI raw current magnet. This dataset contains the raw current values for experiment three investigating the effects of a magnetic constraint.
(CSV)

S5 Dataset E-SOFI current means magnet. This dataset contains the average current values for experiment three investigating the effects of a magnetic constraint.
(CSV)

S6 Dataset E-SOFI velocities raw. This dataset contains the raw velocity measures for experiment four investigating the effects of a magnetic constraint.
(CSV)

S7 Dataset E-SOFI mean velocities. This dataset contains the mean velocity measures for experiment four investigating the effects of a magnetic constraint.
(CSV)

S8 Dataset Simulated voltage bifurcation. This dataset contains data for simulations of the CDM testing for onset of oscillations with varying voltage.
(CSV)

S9 Dataset Simulated amplitude bifurcation. This dataset contains the average amplitude estimates for varying levels of voltage.
(CSV)

## References

[1] Nicolis G. Physics of Far-From-Equilibrium Systems and Self Organization. In: Paul Davies, editor. New Physics Cambridge University Press; 1992.

[2] Kondepudi D, Self-Organization, Entropy Production, and Physical Intelligence. Ecol Psychol. 2012 10.1080/10407413.2012.643716

[3] Eigen M, Schuster P. Berlin The Hyper Cycle 1^st^ Ed Springer; 1979.

[4] Kaufmann S. Oxford Origins of Order 1^st^ Ed Oxford University Press; 1993.

[5] Rosen R. New York Essays on Life Itself 1^st^ Ed Columbia University Press; 2000.

[6] Dixon J, Kondepudi D, Davis T. End-Directedness and Context in Non-Living Dissipative Systems. World Scientific Review. 2015 8 10.1142/9789814730617_0009

[7] Kondepudi D, Kay B, Dixon J. End-Directed Evolution and the Emergence of Energy-Seeking Behavior in a Complex System. Physical Review. 2015 5. 10.1103/PhysRevE.91.050902 26066110

[8] Kondepudi D, Kay B, Dixon J. Dissipative structures, machines, and organisms: A perspective. Chaos. 2017 9; 10.1063/1.5001195 29092452

[9] Jun J. Dynamics of Self-Organization of Ramified Patterns in an Electrochemical System. University of Illinois, Urbana-Champaign 2006.

[10] Belkin A, Hubler A, Bezryadin A. Self-Assembled Wiggling Structures and the Principle of Maximum Entropy Production. Scientific Reports. 2015 10.1038/srep08323 PMC4321171 25662746

[11] Mathis A, Mamidanna P, Cury K, Abe T, Murthy V, Mathis M, et al DeepLabCut: markerless pose estimation of user-defined body parts with deep learning. Nature Neuroscience. 2018 10.1038/s41593-018-0209-y 30127430

[12] Kondepudi D, Prigogine I, Modern Thermodynamics: From Heat Engines to Dissipative Structures 1^st^ Ed John Wiley & Sons; 1998

[13] Prigogine I, Nicolis G, On Symmetry-Breaking Instabilities in Dissipative Systems. The Journal of Chemical Physics. 1967: 46 10.1063/1.1841255

[14] Martyushev L, Seleznev V. Maximum Entropy Production Principle in Physics, Chemistry, and Biology. Physics Reports. 2006: 426 10.1016/j.physrep.2005.12.001

[15] Prigogine I, Introduction to Thermodynamics of Irreversible Processes 2^nd^ Ed Interscience, New York; 1961.

[16] Kleidon A, and Lorentz R D (Eds). Non-equilibrium thermodynamics and the production of entropy. Springer-Verlag, Berlin; 2005.

[17] Endres R. Entropy production selects nonequilibrium states in multistable systems. Scientific Reports. 2017: 7 10.1038/s41598-017-14485-8 29089531 PMC5663838

[18] Kugler P, Turvery M. Self-organization, flow fields, and information. Human Movement Science. 1988: 7 10.1016/0167-9457(88)90009-7

[19] Swenson R, Turvey M. Thermodynamic Reasons for Perception-Action Cycles. Ecological Psychology. 1991 10.1207/s15326969eco0304_2

